# Characterization of Cancer/Testis Antigens as Prognostic Markers of Ovarian Cancer

**DOI:** 10.3390/diagnostics13193092

**Published:** 2023-09-29

**Authors:** Ramilia Vlasenkova, Daliya Konysheva, Alsina Nurgalieva, Ramziya Kiyamova

**Affiliations:** Biomarker Research Laboratory, Institute of Fundamental Medicine and Biology, Kazan Federal University, Kazan 420008, Russia; r.mukhamadeeva@yandex.ru (R.V.);

**Keywords:** cancer-testis antigens, CTA, ovarian cancer

## Abstract

The main goal of this study was to characterize cancer/testis antigens (CTAs) as potential molecular markers of ovarian cancer. First, we gathered and analyzed a significantly large dataset of 21 selected CTAs that are encoded by 32 genes; the dataset consisted of the mutation data, expression data, and survival data of patients with ovarian cancer (n = 15,665). The 19 functionally significant missense mutations were identified in 9 CTA genes: ACRBP, CCT4, KDM5B, MAGEA1, MAGEA4, PIWIL1, PIWIL2, PRAME, and SPA17. The analysis of the mRNA expression levels of 21 CTAs in healthy and tumor ovarian tissue showed an up-regulation in the expression level of AKAP3, MAGEA4, PIWIL1, and PRAME in tumor samples and a down-regulation in the expression level of CTAG1A, CTAG1B, MAGEC1, and PIWIL2. The CCT4 up-regulation and PRAME mutations were correlated with a good prognosis for ovarian cancer, while higher levels of GAGE2A and CT45A1 mRNAs were correlated with a poor prognosis for ovarian cancer patients. Thus, GAGE2, CT45, CCT4, and PRAME cancer/testis antigens can be considered as potential prognostic markers for ovarian tumors, and GAGE2, CCT4, and PRAME were revealed to be correlated with the prognosis for ovarian cancer patients for the first time.

## 1. Introduction

Ovarian cancer is one of the most fatal gynecologic malignancies worldwide, with an estimated incidence of 239,000 cases and 152,000 deaths each year [1]. Currently, treatment options for ovarian cancer are predominantly limited to surgery, radiotherapy, and chemotherapy. Due to the difficulty of achieving an early diagnosis, postoperative tumor recurrence, and late chemotherapy resistance, the 5-year survival rate of advanced ovarian cancer is less than 30% [2]. Hence, there is an urgent need to explore new avenues for early diagnosis, prognosis, and targeted therapy for ovarian cancer.

Tumor immunotherapy restores normal anti-tumor immune responses by restarting and maintaining the tumor–immune cycle, thereby controlling and eliminating tumors [3]. Immunotherapy-targeting tumor antigens has emerged as an ideal option for therapy in ovarian cancer. Among the tumor antigens, the cancer/testis antigen (CTA) has significant immunogenicity and unique expression patterns in humans [3]. CTA is typically expressed in the normal human testis, and a small amount of expression is present in early developing embryos, placenta, and ovaries. Several studies have shown that CTA is overexpressed in various tumor types and is associated with tumor progression [4]. The CTA activation is largely mediated by alterations in DNA methylation [4]. The limited expression of CTAs in normal testis and the high immunogenicity have warranted the identification of new antigens as potential vaccine targets and cancer biomarkers. Of the more than 250 CT antigens described so far [5], those encoded on the X chromosome (CT-X) are the most CT-limited and most immunogenic in cancer patients. Among CTAs, the melanoma A-3 antigen family (MAGE A3) and cancer/testicular antigen 1B (CTAG1B, best known as NY-ESO-1) have been specifically targeted for therapeutic interventions in many completed and ongoing clinical trials as recombinant protein vaccines [6]. It should be noted that the cancer/testis antigen-45 family (CT45) and Sp17 are also prospective targets of immunotherapy [7]. The diagnostic potential of CTAs, specifically NY-ESO-1, was recently confirmed by studies on synovial sarcoma and liposarcoma [8,9]. This CTA was previously found to be expressed by 80% of synovial sarcomas [10]. In addition, emerging data show that the overexpression of oncogenic CTAs increases the stemness of tumor cells; increases the oncogenicity of cancer cells, motility, metastasis and drug resistance; and, as a rule, is associated with a poor prognosis in cancer patients [11]. To date, the role of CTA as diagnostic, prognostic, and predictive markers of ovarian cancer has not been fully studied. To better understand the prognostic potential of CTAs in ovarian cancer, the thorough analysis of ovarian cancer-associated CTAs using open data sources such as TCGA and ICGC databases is needed.

In order to consider CTAs as potential prognostic markers in ovarian cancer, we performed an analysis of the mutation and expression of selected ovarian cancer-associated CTAs and evaluated their impact on the life expectancy of ovarian cancer patients from studies that are publicly available online.

## 2. Materials and Methods

### 2.1. Data Collection and Preparation

Four open-access databases were used to collect the data: TCGA—The Cancer Genome Atlas; AACR—Project Genie; ICGC—The Insertional Cancer Genome Consortium; and ArrayExpress (data downloaded on 15 May 2021). Data on mutations and gene expression were gathered for the CTA genes. The cBioPortal platform [12] was used to access the data from the 3 TCGA studies of ovarian carcinomas, which consisted of data on gene mutations, gene expression levels, and clinical data. The AACR Project Genie database that was also based on the cBioPortal platform [13] was utilized to obtain 1 study of ovarian carcinoma, which consisted of only gene mutation data. From the ICGC database [14], 2 studies of ovarian carcinomas were used to obtain data on gene mutations, gene expression, and clinical data. From the ArrayExpress database, the E-MTAB-3732 study [15,16] was used to obtain the expression levels of CTAs from tumor and relatively healthy tissue samples (ovarian surface epithelial cells) of ovarian tissue. The summary can be found in Table 1.

### 2.2. Prediction of the Functional Impact of Mutation in CTA Genes

The analysis was conducted on the CTA mutational data from three databases: The Cancer Genome Atlas, ICGC, and AACR Project Genie. Using tools for the prediction of the functional significance of gene mutations, missense and indel (insertion or deletion) CTA gene mutations were examined. Missense mutations were evaluated using the following tools: PROVEAN [17], SIFT [18], PolyPhen-2 [19], Panther-PSEP [20], FATHMM [21], REVEL [22], Mutation Taster [23], and Mutation Assessor [24]. A missense mutation had to be detected by at least four of the tools during the prediction of the functional significance of mutations in order to be classified as pathogenic. The PROVEAN tool was used to analyze indel mutations. Using the Conserved Domains and Protein Classification resource [25], highly conserved regions of the CTA protein were identified.

### 2.3. Analysis of the Expression Levels of CTA mRNAs

Using the ArrayExpress CTA expression data, a comparison of the gene expression levels of CTAs in relatively healthy and tumor tissue samples was conducted. The comparison of the tumor and healthy tissue samples was performed using the Wilcoxon test (*p* < 0.05).

### 2.4. Analysis of the Co-Expression Networks of CTA mRNAs

The analysis and visualization of co-expression networks of CTA mRNAs was carried out using the tidygraph [26] and ggraph packages [27]. The cut-off for the selection of hub genes in the networks was 0.8.

### 2.5. Survival Analysis of CTAs

The Kaplan–Meier estimate was applied in a survival analysis (*p* < 0.05). According to the level of CTA mRNA expression (up-regulation was defined as two standard deviations above the mean of the samples in each dataset) and the presence or absence of a CTA gene mutation, the tumor samples were divided into groups.

A multivariate survival analysis using Cox regression was carried out for each study, in which we discovered significant differences between the patient groups by using the Kaplan–Meier estimate (*p* < 0.05). This analysis was carried out to determine if one of the key factors affecting a patient’s likelihood of survival was the level of CTA expression or the CTA mutations. We used the clinical parameters that were available for each study in this analysis.

## 3. Results

### 3.1. Selection of Ovarian Cancer-Associated CTAs

At the beginning, several ovarian cancer-associated CTA proteins were determined by conducting a literature search and by using the cBioPortal integrated survival analysis tool.

According to the literature analysis, to date, 38 CTAs have been associated with ovarian cancer [3]. The CTAs of interest were selected by several criteria, including the presence of (1) mRNA expression of CTAs in ovarian cancer samples, (2) protein expression of CTAs in ovarian cancer samples [28], and (3) expression of CTAs in the ascitic fluid of ovarian tumor cancer patients [3]. In addition, by using the cBioPortal integrated survival analysis tool, we selected CTAs whose mRNA expression or mutation correlated with the survivability of ovarian cancer patients (4). The dataset for survival analysis at this stage only included 585 samples. Therefore, we selected the CTAs that had at least one of the four characteristics under consideration. Overall, we selected 21 out of 38 CTAs for further analysis, which were encoded by 32 genes; this is because several CTAs, such as CT45, NY-ESO-1, and GAGE1/2, are encoded by more than one gene (Table 2).

### 3.2. Dataset Description

From four databases (TCGA, Genie, ICGC, and ArrayExpress), we gathered the mutational and expression data of selected CTAs as well as the clinical data from six ovarian cancer studies. There were 15,665 samples collected in total. The summary can be found in Table 1.

### 3.3. Evaluation of the Functional Impact of Single Nucleotide Polymorphisms in 32 Selected Genes of 21 CTAs

The samples from three databases (TCGA, *n* = 606; Genie, *n* = 4230, ICGC, *n* = 88) revealed 79 mutations in 14 out of the 32 genes of 21 CTAs, including 62 missense mutations, 7 nonsense mutations, and 10 same-sense mutations.

By using tools for the prediction of the functional significance of mutations, including PROVEAN, SIFT, PolyPhen-2, Panther-PSEP, FATHMM, REVEL, Mutation Taster, and Mutation Assessor, 19 functionally significant missense mutations in 9 CTA genes were found out of the entire mutational dataset. Of all functionally significant mutations, six mutations are located in highly conserved gene regions; these mutations are Pro428Thr in the CCT4 gene, Leu741Ile in the KDM5B gene, Tyr79Cys in the ACRBP gene, Val694Met and Gly727Asp in the PIWIL2 gene, and Gly241Trp in the MAGEA4 gene. Table 3 displays the list of functionally significant mutations, minor allele frequencies (MAFs), and rsIDs (identification numbers from the dbSNP database), as well as the indication of highly conserved regions.

### 3.4. Comparison of the mRNA Expression Levels of 32 Genes of Selected CTAs between Healthy and Tumor Ovarian Tissue

The ArrayExpress E-MTAB-3732 study (*n* = 573) provided the CTA expression data that was used for the comparison between tumor tissue samples and relatively healthy (ovarian surface epithelial cells) tissue samples. It should be pointed out that the methylation information that could affect the expression of CTAs was not present in the data that we used, so we did not account for it in our analysis.

**Figure 1:**
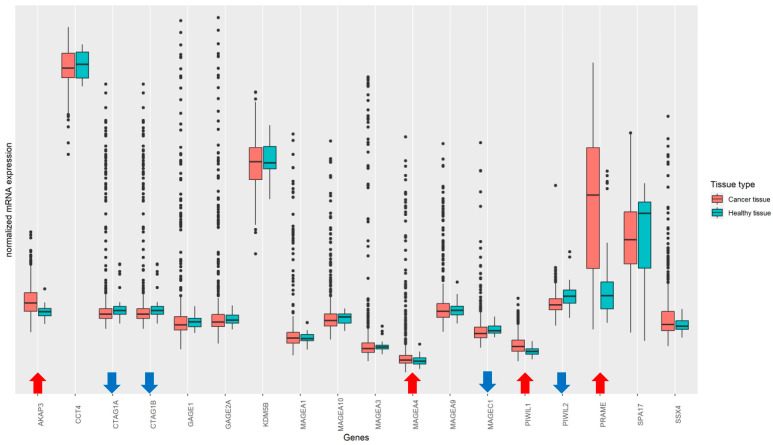
mRNA expression levels of 32 genes that encode 21 selected CTAs in healthy and tumor ovarian tissue (ArrayExpress database, *n* = 573, Wilcoxon test (*p* < 0.05)). Up-regulation in the expression level of the AKAP3 (*p* < 0.0001), MAGEA4 (*p* = 0.043), PIWIL1 (*p* < 0.0001), and PRAME (*p* = 0.043) genes was found in tumor samples; down-regulation in tumors was detected for the CTAG1A (*p* = 0.012), CTAG1B (*p* = 0.013), MAGEC1 (*p* = 0.004), and PIWIL2 (*p* < 0.0001) genes.

The comparison of the levels of the mRNA expression of 32 genes encoding 21 selected CTAs between ovarian relatively healthy and ovarian tumor tissue were conducted using the Wilcoxon test (*p* < 0.05). Up-regulation in the expression level of the AKAP3 (*p* < 0.0001), MAGEA4 (*p* = 0.043), PIWIL1 (*p* < 0.0001), and PRAME (*p* = 0.043) genes was found in the tumor samples, and lower levels of expression were detected in tumors for the CTAG1A (*p* = 0.012), CTAG1B (*p* = 0.013), MAGEC1 (*p* = 0.004), PIWIL2 (*p* < 0. 0001) genes (Figure 1).

### 3.5. Comparison of the Co-Expression Networks of 32 Genes of Selected CTAs mRNAs between Healthy and Tumor Ovarian Tissue

**Figure 2:**
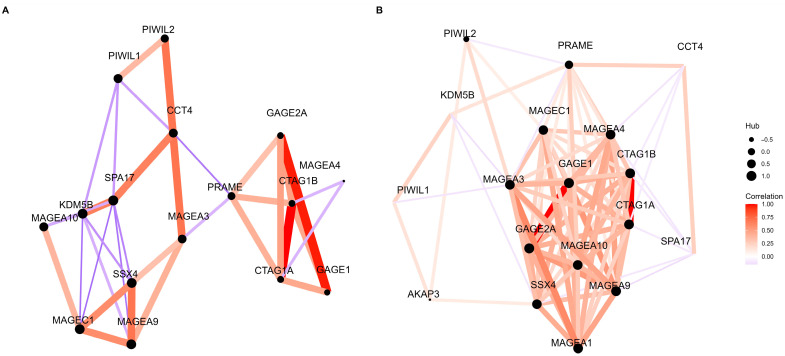
Co-expression network of 32 genes that encode 21 selected CTAs in healthy (**A**) and tumor ovarian (**B**) tissue (ArrayExpress database, *n* = 573).

The analysis of the co-expression networks of 32 genes that encode 21 selected CTAs showed different patterns of co-expression in healthy (ovarian surface epithelial cells) and tumor ovarian tissue (Figure 2). In healthy ovarian tissue samples, there were no negative correlations between CTAs, but there were negative correlations in tumor samples.

Moreover, we identified hub genes in the co-expression networks of 32 CTA mRNAs. For healthy ovarian tissue samples, five hub genes were identified: SPA17, MAGEC1, KDM5B, SSX4, and MAGEA9. For ovarian tumor samples, we detected six hub genes: GAGE2A, GAGE1, MAGEA9, CTAG1A, CTAG1B, and MAGEA1.

### 3.6. Characterization of the 21 Selected Ovarian Cancer-Associated CTAs as Potential Ovarian Cancer Prognostic Markers

Using the Kaplan–Meier estimator, a survival analysis of the ovarian carcinoma datasets from the TCGA, ICGC, and GENIE studies was performed. Two criteria were used for grouping the tumor samples: the expression level of the 21 CTA mRNAs and the presence or absence of a CTA gene mutation. The dataset included 15,665 samples.

The survival analysis (including the overall and disease-free survival analysis) revealed a significant correlation of the mutation profile with the life expectancy of patients for the CTA gene PRAME and a significant correlation of mRNA expression with the life expectancy of patients for three CTA genes, including CCT4, CT45A1, and GAGE2A.

**Figure 3:**
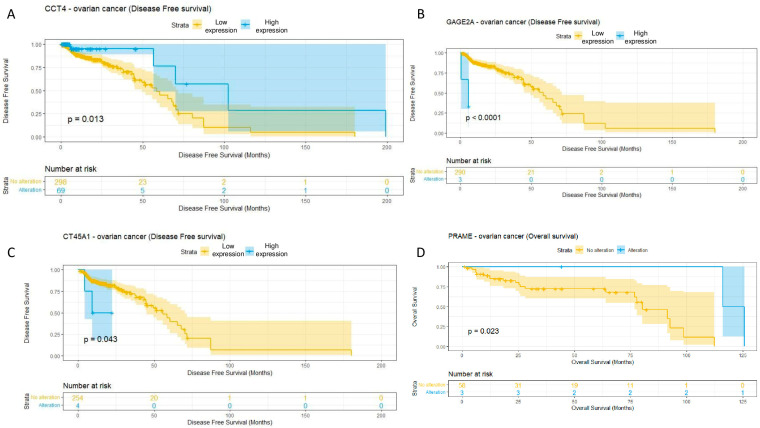
Survival analysis using the mRNA expression and mutational profiles of 4 genes that encode the corresponding CTAs. (**A**) CCT4 expression (*p*-value = 0.013, *n* = 367), (**B**) GAGE2A expression (*p*-value < 0.0001, *n* = 293), (**C**) CT45A1 expression (*p*-value = 0.043, *n* = 258), (**D**) PRAME mutations (*p*-value = 0.023, *n* = 61). Expression data sources: TCGA, ICGC. Mutational data sources: TCGA, ICGC, GENIE.

Thus, disease-free survival was significantly higher in the group of patients with increased expression of the CCT4 gene in the ovarian cancer samples (Figure 3A; *p*-value = 0.013, *n* = 367). On the contrary, in patients with an increased expression in the GAGE2A (Figure 3B; *p*-value < 0.0001, *n* = 293) and CT45A1 genes (Figure 3C; *p*-value = 0.043, *n* = 258), disease-free survival was significantly lower. The presence of mutation in the PRAME gene correlates with a good prognosis (Figure 3D; *p*-value = 0.023, *n* = 61).

**Figure 4:**
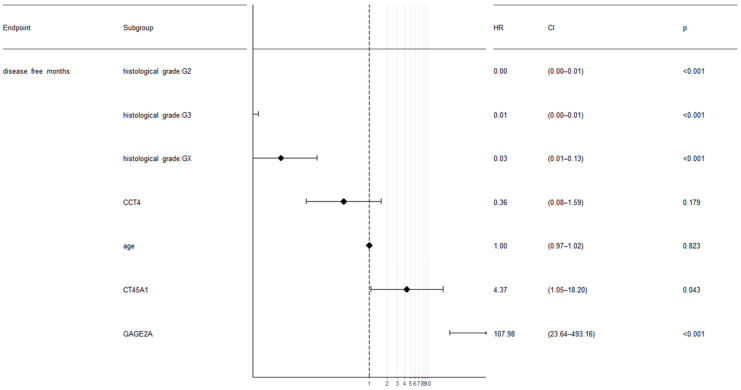
Multivariate disease-free survival analysis based on clinical parameters and mutational and mRNA expression profiles of three genes: CCT4, CT45A1, and GAGE2A (TCGA, ICGC, *n* = 437).

The correlation of increased expression levels of CT45A1 and GAGE2A genes with a reduced life expectancy (disease-free survival) was also confirmed by a multivariate survival analysis (Figure 4).

## 4. Discussion

According to the literature, to date, there are 38 CTAs that are associated with ovarian cancer [3]. To select the CTAs for our further analysis, we performed an analysis of the mRNA and protein expression of 38 ovarian cancer-associated CTAs in ovarian cancer samples [28] as well as the expression of these CTAs in the cells of ascitic fluid of ovarian tumors [3] using a literature search. Moreover, an analysis of the correlation of mRNA expression and/or mutation in CTAs with the survivability of ovarian cancer patients was performed using cBioPortal data, which included 585 samples. Overall, 21 of the CTAs encoded by 32 genes that had at least one of the characteristics under consideration were selected for further analysis.

We analyzed the 21 selected CTAs’ mutational data, the mRNA expression data, and the survival data of ovarian cancer patients in correlation with the mutational and expression data of selected CTAs in order to determine the potential role of CTAs as prognostic markers of ovarian cancer. At this stage, the dataset numbered more than 15,000 samples, and by doing so, we increased the initial dataset by 25 times.

The analysis of mutations in the 32 genes of the 21 CTAs revealed 79 mutations in 14 genes: 62 missense mutations, 7 nonsense mutations, and 10 same-sense mutations. We identified 19 functionally significant missense mutations in 9 CTA genes, including in ACRBP, CCT4, KDM5B, MAGEA1, MAGEA4, PIWIL1, PIWIL2, PRAME and SPA17. It should be noted that six mutations were located in highly conserved gene regions of the CTA genes: ACRBP—Tyr79Cys; CCT4—Pro428Thr; KDM5B—Leu741Ile; MAGEA4—Gly241Trp; and PIWIL2—Val694Met and Gly727Asp.

The comparison of the mRNA expression levels of 32 genes of the 21 CTAs between relatively healthy and tumor ovarian tissue showed up-regulation in the expression level of the AKAP3, MAGEA4, PIWIL1, and PRAME genes in tumor samples. It was shown that higher levels of AKAP3 expression were associated with a poor survivability of ovarian cancer patients [29]. Previously, high expressions of MAGEA4 and PIWIL1 were found in epithelial ovarian cancer [30,31]; furthermore, the expression of PRAME was found in ovarian, uterine, and other tumor types [32]. Lower levels of mRNA expression of genes of the 21 CTAs in ovarian tumors were detected for CTAG1A, CTAG1B, MAGEC1, and PIWIL2. To examine patterns of expression in tumor and healthy ovarian tissue and find interconnected genes across the selected CTAs, the analysis of co-expression networks of CTA mRNAs was conducted. The examination indicated the hub genes in tumor samples, including GAGE2A, GAGE1, MAGEA9, CTAG1A, CTAG1B, and MAGEA1; and the hub genes in healthy ovarian tissue samples, including SPA17, MAGEC1, KDM5B, SSX4, and MAGEA9. It should be noted that only the MAGEA9 gene was common for the co-expression networks of CTAs mRNAs among tumor and healthy ovarian tissue. Different expression profiles of CTAs in tumor and healthy ovarian tissue reflect global demethylation processes that occur during malignant transformation in ovarian cells [4].

A survival analysis using the Kaplan–Meier estimator showed the potential impact of GAGE2A and CT45A1 up-regulation on poor disease-free survival prognosis in ovarian tumors, which was confirmed by a multivariate survival analysis. Aside from that, CCT4 up-regulation and PRAME mutations was correlated with a good prognosis for ovarian cancer patients. One of the mutations in the PRAME gene that correlated with survivability of ovarian cancer patients was the functionally significant Ala99Ser mutation. Interestingly, the GAGE2, CCT4, and PRAME cancer/testis antigens were revealed to be correlated with the prognosis for ovarian cancer patients for the first time, while a high expression of the CT45 cancer/testis antigen was already identified as a prognostic factor in ovarian tumors [33,34] as well as in other cancer types, such as lung cancer, breast cancer, gastric tumors, myeloma, Hodgkin’s lymphoma, and fibrosarcoma [10,35,36,37,38,39]. Apart from that, it was previously shown that PRAME was associated with an advanced tumor grade and poor prognosis in breast, cervical, hepatic, prostate, bladder, and other types of cancer [4,40]. Moreover, CCT4 were considered as a promising biomarker for the prognosis of head and neck squamous cancer [41].

In conclusion, we found 38 ovarian cancer-associated CTAs using a literature search and chose 21 of them for further analysis. Among the 21 selected CTAs, GAGE2, CT45, CCT4, and PRAME cancer/testis antigens were revealed to be correlated with the prognosis for ovarian cancer patients, and GAGE2, CCT4, and PRAME were identified for the first time. GAGE2 was also identified as the hub gene in the co-expression networks of ovarian tumor samples. Thus, GAGE2, CT45, CCT4, and PRAME cancer/testis antigens can be considered as new potential prognostic markers for ovarian cancer disease and need to be further investigated.

## Figures and Tables

**Table 1:** Summary of the gathered dataset representing the sample count for every database.

Database	Study	Mutation Data	Expression Data	Survival Data
cBioPortal, TCGA	Firehose Legacy (*n* = 617)	83	62	28
	Nature 2011 (*n* = 489)	316	189	488
	PanCancer (*n* = 585)	207	300	86
cBioPortal, AACR	Genie v9.0, (*n* = 6097)	4230	-	-
ICGC	OV-US and OV-AU (*n* = 15,063)	88	1677	15,063
ArrayExpress	E-MTAB-3732 (*n* = 573)	-	573	-
Summary		4924	2801	15,665

**Table 2:** The list of 21 ovarian cancer-associated CTAs, which were selected according to four criteria, including the mRNA expression of CTAs in ovarian cancer samples, protein expression of CTAs in ovarian cancer samples, expression of CTAs in ascitic fluid of ovarian tumor cancer patients, and mRNA expression or mutation correlated with survivability of ovarian cancer patients. For the expression section of the table, ‘+’ means 1%–25% of samples, ‘++’ means 25–50% samples, and ‘+++’ means 50–75% samples; for the survival analysis, ‘+’ means *p*-value < 0.1, ‘++’ means *p*-value < 0.05, and ‘+++’ means *p*-value < 0.01. * The NAPI2B protein encoded by SLC34A2 gene was used as a control because its expression level and mutations are known markers of ovarian cancer.

CTA Name	Genes	Expression		Presence	Survival Depends On
		(1) mRNA (*n* > 50)	(2) Protein (*n* > 50)	(3) In Ascitic Fluid	(4) Expression and\or Mutations
CT45	CT45A1-CT45A3, CT45A5-CT45A10	+	++	-	-
NY-ESO-1	CTAG1A, CTAG1B	++	++	-	+
PIWIL2	PIWIL2	++	-	-	-
OY-TES-1	ACRBP	++	+++	-	+
MAGE-A4	MAGEA4	++	+	-	-
MAGE-A1	MAGEA1	++	+	+	-
MAGE-A3	MAGEA3	++	++	++	-
AKAP3	AKAP3	++	-	-	-
PRAME	PRAME	+++	-	-	-
PIWIL1	PIWIL1	+++	-	-	-
MAGEC1	MAGEC1	-	+	-	-
MAGEA9	MAGEA9	-	++	-	-
SP17	SPA17	-	++	-	-
PLU-1	KDM5B	++	-	-	-
GAGE 1/2	GAGE1, GAGE2A, GAGE2C	-	-	++	-
BAGE	BAGE	-	-	++	-
MAGEA10	MAGEA10	-	-	-	++
CCT4	CCT4	-	-	-	+++
PIWIL4	PIWIL4	-	-	-	+++
GAGE 4	GAGE4	-	-	-	+
SSX4 NAPI2B *	SSX4 SLC34A2	- +++	- +++	- +++	+ +++

**Table 3:** Functionally significant missense mutations in 14 out of 32 selected CTA genes detected in the ovarian tumor samples (TCGA: 13 samples; ICGC: 7 samples; GENIE: 3 samples). The ‘+’ sign in the ‘Conserved Region’ column means that the mutation is located in the conserved region of the protein.

Gene	Mutation	rsIDs	MAF	Conserved Region
ACRBP	Gly417Arg	rs577478690	0.000007968	
ACRBP	Gln532His	-	-	
ACRBP	Tyr79Cys	-	-	+
CCT4	Glu459Asp	-	-	
CCT4	Ile529Val	-	-	
CCT4	Pro428Thr	-	-	+
KDM5B	Leu741Ile	-	-	+
KDM5B	Arg1543Gln	rs761912360	0.0000531	
KDM5B	Arg380Leu	-	-	
MAGEA1	Lys278Ile	-	-	
MAGEA1	Arg118Gly	rs373351745	0.0000146	
MAGEA1	Val309Asp	-	-	
MAGEA4	Gly241Trp	-	-	+
MAGEA4	Ile151Asn	-	-	
PIWIL1	Cys266Phe	-	-	
PIWIL2	Gly727Asp	-	-	+
PIWIL2	Val694Met	rs778216689	0.00001171	+
PRAME	Ala99Ser	-	-	
SPA17	Arg126Trp	-	-	

## Data Availability

Publicly available datasets were analyzed in this study. These datasets can be found here (the data were downloaded on 15 May 2021): http://www.cbioportal.org, https://genie.cbioportal.org/, https://dcc.icgc.org/, and https://www.ebi.ac.uk/arrayexpress/experiments/E-MTAB-3732.
